# Regulatory network operations in the Pathway Tools software

**DOI:** 10.1186/1471-2105-13-243

**Published:** 2012-09-24

**Authors:** Suzanne M Paley, Mario Latendresse, Peter D Karp

**Affiliations:** 1 Bioinformatics Research Group, SRI International 333 Ravenswood Ave, Menlo Park, CA 94025

**Keywords:** Regulatory networks, Regulatory interactions, Regulation ontology, Bioinformatics

## Abstract

**Background:**

Biologists are elucidating complex collections of genetic regulatory data for multiple organisms. Software is needed for such regulatory network data.

**Results:**

The Pathway Tools software supports storage and manipulation of regulatory information through a variety of strategies. The Pathway Tools regulation ontology captures transcriptional and translational regulation, substrate-level regulation of enzyme activity, post-translational modifications, and regulatory pathways. Regulatory visualizations include a novel diagram that summarizes all regulatory influences on a gene; a transcription-unit diagram, and an interactive visualization of a full transcriptional regulatory network that can be painted with gene expression data to probe correlations between gene expression and regulatory mechanisms. We introduce a novel type of enrichment analysis that asks whether a gene-expression dataset is over-represented for known regulators. We present algorithms for ranking the degree of regulatory influence of genes, and for computing the net positive and negative regulatory influences on a gene.

**Conclusions:**

Pathway Tools provides a comprehensive environment for manipulating molecular regulatory interactions that integrates regulatory data with an organism’s genome and metabolic network. Curated collections of regulatory data authored using Pathway Tools are available for Escherichia coli, Bacillus subtilis, and *Shewanella oneidensis*.

## Background

Cells have evolved multiple molecular regulatory modalities. For example, in addition to having its activity regulated directly by a ligand, an enzyme can be regulated at the point of transcription, translation or degradation. It can be sequestered or covalently modified. And all of these processes can themselves be subject to regulation.

Here we report our progress in developing a comprehensive environment for capturing, interrogating, visualizing, and computing with individual regulatory interactions, and with regulatory networks. Currently this environment emphasizes prokaryotic rather than eukaryotic regulatory mechanisms. At the core of our efforts is a regulation ontology for capturing regulatory interactions in a declarative, computable fashion. A set of interactive editing tools allows curation of regulatory interactions and the molecules they regulate. We have also developed computational tools for interrogating and displaying individual regulatory interactions, and genome-scale regulatory networks.

These tools have been implemented in the Pathway Tools software [1], which is a comprehensive systems-biology software environment for management, analysis, and visualization of integrated collections of genome, pathway, and regulatory data. It supports creation, curation, dissemination and Web-publishing of organism-specific databases, called Pathway/Genome Databases (PGDBs), that integrate many types of data. It performs computational inferences, including prediction of metabolic pathways, prediction of metabolic pathway hole fillers, and prediction of operons. The software also supports the development of metabolic-flux models using flux-balance analysis [2].

All of the software and data features described in this paper are available in version 16.0 of the Pathway Tools software, with the exception of the tool described in Section Inferring regulatory influences on a gene, which currently exists as a research prototype only. Because this paper attempts to summarize all the regulation-related features in Pathway Tools, it includes some components that have been part of the software for some time. Table 1 highlights those features that are new since [1]. In addition, the amount of regulatory data represented in EcoCyc, BsubCyc and other PGDBs has increased substantially.

**Table 1:** Major new features

Ontology classes	Compound-Mediated-Translation-Regulation (riboswitches)
	Allosteric-Regulation-of-RNAP
Visualizations	Regulation Summary Diagram (section The regulation summary diagram)
	Specialized Signaling Pathway display and editing (section Pathway diagrams)
	Port of Regulatory Overview (section Regulatory overview) to BioCyc website
Computational Analyses	Object Group Operations and Regulation Enrichment Analysis (section Object group operations and regulation-enrichment analysis) Export of Regulatory Network
	to XGMML (Cytoscape) (section Export to cytoscape) Ranking Genes According
	to Regulatory Influence (section Ranking genes according to regulatory influence)
	Web services access to regulatory data

Regulation-related features or capabilities described in this paper that are new in Pathway Tools since the last major Pathway Tools paper [1].

## Implementation

The implementation of the regulation operations within Pathway Tools follow the same implementation approach as described in [1].

## An ontology of regulatory interactions

The Pathway Tools schema (ontology) organizes biological information in a structured fashion, so that data can be made readily accessible for computational analysis. The ontology is designed to enable high-fidelity representation of regulatory relationships. It is also designed to represent incomplete information (e.g., we might know that a given transcription factor controls all the genes within an operon without knowing the location of the promoter for that operon). Currently, the ontology is qualitative: it does not capture quantitative information about regulation.

**Figure 1:**
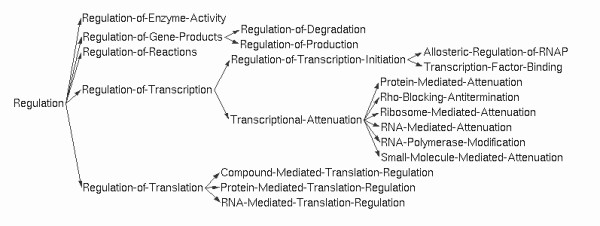
The Regulation class hierarchy.

The Pathway Tools schema is organized into a class hierarchy. Each class has a set of slots that define the attributes and relationships of instances of those classes. Classes inherit slots from their parent classes. Most forms of regulation are collected under the class Regulation, which represents a single molecular regulatory interaction. The Regulation class is a root class in the ontology, that is, it has no parents. Figure 1 shows the tree of subclasses under the Regulation class.

The Regulation class defines several relationship slots that are inherited by all of its subclasses and instances. The slot Regulator specifies the regulator object in the regulatory interaction (such as a protein or a small molecule). The slot Regulated-Entity specifies the object whose activity is being regulated (such as a gene, a transcription unit (TU) ^a^, a reaction, or a catalysis object). The slot Mode indicates whether the regulation is positive (activating), negative (inhibitory) or unknown. Subclasses of the Regulation class define additional slots specific to those types of regulatory interactions. A few of the major subclasses are described below. 

• Regulation of Enzymatic Activity: This class defines substrate-level modulation of an enzyme. Its Mechanism slot indicates whether regulation is allosteric, competitive, etc. Because many purely *in vitro* activators and inhibitors are reported in the literature, an additional slot indicates whether or not the regulation is physiologically relevant *in vivo*.

• Transcription Factor Binding: This class represents the binding of a regulator to a DNA binding site in order to regulate the binding of RNA polymerase to a promoter and subsequent transcription. The regulator is the transcription factor — when the ligand that activates or deactivates the transcription factor is known, that information is indicated by specifying as the regulator the database object representing the appropriate chemically modified form of the transcription factor. An additional slot, Associated-Binding-Site, provides a link to the binding site. The regulated entity here is the promoter object. The process of transcription is not explicitly represented in the Pathway Tools schema. Rather, regulation of a promoter implies regulation of all genes in the TU governed by that promoter (the promoter object indicates the sigma factor that recognizes that promoter).

• Transcriptional Attenuation: Attenuation is the premature termination of transcription. In most cases, the presence or absence of a regulator determines whether the mRNA secondary structure of the attenuator region forms a terminator or anti-terminator structure. Only genes downstream from the potential terminator are regulated by attenuation. Thus for this class, we consider the terminator to be the regulated entity: it is implicit that regulation of a terminator affects all downstream genes in the same TU. The Regulation of Attenuation class contains six subclasses, each describing a different mechanism of attenuation. Some subclasses have additional slots for the genome coordinates of the anti-terminator (and anti-anti-terminator). The Ribosome-Mediated Attenuation subclass has a slot for the ribosome pause site, whereas other subclasses have a slot that identifies the mRNA binding site recognized by the regulator.

•Regulation of Translation: Like transcription, neither the process of translating mRNA to protein, nor mRNA itself, are explicitly represented in the Pathway Tools schema. Rather, the Regulated-Entity for translational regulation is the TU (a collection of genes transcribed together, governed by a single promoter) or individual gene. In general, regulation of translation occurs either by blocking or unblocking the binding of the ribosome (directly or indirectly), or by stabilizing or destabilizing the mRNA, thereby governing whether or not it can be translated before it is degraded. Because these two mechanisms often occur in concert, we did not define separate subclasses for them. Rather, the Mechanism slot indicates whether the regulation is by ribosome-blocking, mRNA-degradation or both. The Associated-Binding-Site slot links to the mRNA binding site for the regulator. Subclasses of the Regulation of Translation class allow for additional slots. For example, the RNA-Mediated Translation Regulation class includes slots that identify accessory proteins and associated RNases for a given interaction.

Some proteins are regulated, not by any of the mechanisms described above, but by post-translational modifications, ligand-binding, or sequestering in various ways. Rather than try to model these phenomena using children of the Regulation class, we represent these interactions explicitly as individual reaction objects.

The use of the Regulation class can be considered a level of abstraction above that of using individual reactions. We could have chosen instead to explicitly represent each of the phenomena above as individual reactions; for example, explicitly modeling the binding of a transcription factor to its binding site or the conversion of one RNA secondary structure to another as reactions. How do we decide when it is preferable to represent a regulatory phenomenon as discrete reactions and when to simplify by using a Regulation frame? A Regulation frame represents a biological idiom — it is shorthand for a set of largely stereotyped interactions in which most of the details remain the same in each example, with only a few key differences (e.g., the identity of the regulator or regulated entity, the location of the binding site). By using such idioms, we not only reduce the complexity of the model, making it easier to understand and manipulate, but we also highlight commonalities that might not otherwise be obvious. For example, the complete set of interactions that would be needed to represent regulation of transcription would look very different from the set of interactions needed to represent regulation of translation. By modeling both as Regulation objects, however, it becomes clear that the most important aspect is the presence or absence of the regulator impacts whether or not a particular protein is produced.

However, the idiom becomes less useful when it is not appreciably simpler than the underlying interactions, or when it costs too much in the way of representational power. It is no simpler to say that one protein inhibits another by sequestering it in a complex than to represent the formation of the complex as a reaction; by representing the reaction explicitly, we can then incorporate it into a larger signaling pathway when appropriate. Thus, the boundaries are not always clear-cut; for example, competitive inhibition of an enzyme could be modeled relatively simply as competing reactions of the enzyme with its substrate or its inhibitor, but since enzyme modulation is a fairly well-understood idiom, and because we have chosen not to explicitly model the formation of the enzyme-substrate complex, we use a Regulation object for this kind of interaction. We use the regulation abstraction when we consider its value to outweigh its cost. The end result is that to determine the full set of regulatory influences on a given protein, we must consider the Regulation objects that affect it or its gene, and the reactions in which it participates.

Our representation of regulation is still a work in progress. For example, although we have created a class for regulation of protein degradation, it has not yet been fleshed out with the details a curator might wish to capture. Nor can we yet represent regulation in which the regulator is an environmental condition (e.g., temperature, pH) instead of a protein or small molecule. We expect to address these issues in the future.

## Regulation data content in BioCyc

**Figure 2:**
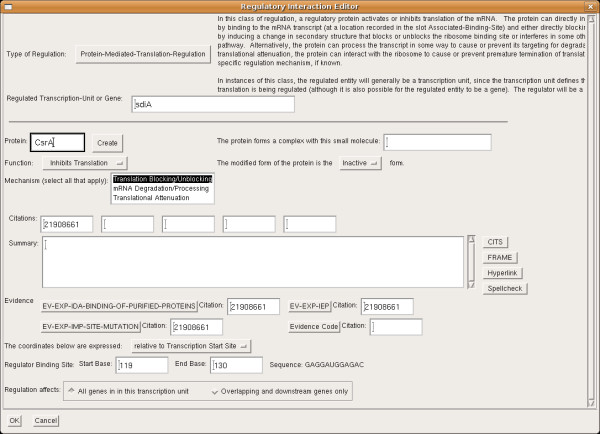
A sample regulatory interaction editor dialog.

Pathway Tools contains a full suite of form-based interactive editors for curation of regulation. The regulatory interaction editor allows the user to specify the class of regulation, the object being regulated, the regulator, and whether the regulation is activation or inhibition. There are fields for entering literature citations, evidence codes for the regulatory interaction, and a textual commentary. Depending on the class of regulation, additional fields enable the user to specify a binding site location, whether or not a protein regulator has a small molecule ligand, a mechanism, and/or other sites of interest, such as the anti-terminator and pause sites for attenuation interactions. An example editor is shown in Figure 2.

The regulation editors have been used for entry of regulatory interactions by several projects shown in Table 2 that utilize Pathway Tools, for E. coli (EcoCyc [3] v.16.0), B. subtilis (BsubCyc v.16.0, developed by Ingrid Keseler at SRI), *S. oneidensis* (hosted at Shewanella Knowledgebase [4]), and *Synechococcus* (curated by Margrethe Serres at the Marine Biological Laboratory, but not yet publically available.) In some cases we have also written programs to import data in bulk from various sources. Note that transcriptional regulation data is shared between EcoCyc and RegulonDB [5] (the data are curated into EcoCyc and periodically transferred to RegulonDB); the two complements of regulatory data are largely identical. Data regarding transcriptional regulation in BsubCyc have been imported from the Database of Transcriptional Regulation in B. subtilis (DBTBS) [6].

**Table 2:** Selected statistics on regulation data content for several organisms

	***E. coli *K-12**	***B. subtilis***	***S. oneidensis***	***Shewanella *sp.**	***Synechococcus***
	**MG1655**	***subtilis*168**	**MR1**	**W3-18-1**	**sp. PCC7002**
Transcriptional regulation	3118	781	589	569	114
Transcription factor binding	3032	747	589	569	114
Allosteric regulation of RNA-polymerase	66	0	0	0	0
Attenuation	20	34	0	0	0
Translational regulation	97	5	0	0	4
Protein-mediated	31	2	0	0	0
RNA-mediated	59	1	0	0	0
Small-molecule-mediated	5	2	0	0	4
Enzyme modulation	2508	9	0	0	5
Physiologically relevant	210	2	0	0	2
Genes with ≥ 1 transcriptional/translational regulator	1771	1087	542	539	124
Percent of genome	39%	25%	12%	13%	4%
Transcriptional or translational regulators	260	151	62	54	19
Proteins	225	128	62	54	16
RNAs	27	15	0	0	0
Small molecules	8	8	0	0	3
Enzymes subject to modulation	597	8	0	0	3
Physiologically relevant^a^	114	1			1
Enzyme modulators	1016	6	0	0	5
Physiologically relevant^a^	108	2			2

^a^For enzyme modulation, a distinction is made between physiologically relevant effects and those that have been observed purely *in vitro*.

## Visualization of regulation data

Because different users are interested in different aspects of regulation, we have found that there is no single visualization that best captures all regulation data. Some users may be primarily interested in the local effects of operon structure on transcription or translation, whereas others are interested in a wider view of all the regulatory effects on a protein, possibly including indirect regulators. Still others may be more interested in a pathway-based or an organism-wide view of regulation. Thus, we have developed a range of visualizations, each with a different focus and a different level of detail. All of our diagrams except for signaling pathway diagrams are computationally generated based on queries to the regulatory interactions in a given PGDB.

### The transcription unit diagram

**Figure 3:**
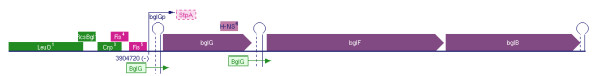
**The transcription unit diagram for the *E. coli *bglGFB operon, showing regulation by a variety of transcription factors and attenuation by BglG.** The diagram is not to scale; rather, the regulatory region has been expanded to show detail with the genes compacted. Sites shown in green activate and sites shown in magenta inhibit transcription. The location of the StpA binding site is unknown.

The TU diagram (see Figure 3) depicts a set of co-transcribed genes in order on the chromosome, and shows the relative positions of the transcription start site and all DNA binding sites that regulate the promoter (not necessarily to scale). It also shows relevant mRNA binding sites, riboswitches, and attenuators. Mousing over a particular binding site brings up a tooltip containing additional information, including the evidence for that binding site. If a gene is part of multiple TUs (as a result of multiple promoters), the gene display page for that gene includes a separate diagram for each TU to allow the user to see which regulatory elements influence which promoter(s). Similarly, the display page for a transcription factor or other regulator includes TU diagrams for all genes regulated by that transcription factor or regulator. Clicking on a promoter arrow in a TU diagram navigates to a TU display page, which contains further details about each regulatory interaction in that TU, including transcription factor ligands, binding site sequences, and the position and sequence of different attenuation elements (e.g., the terminator, anti-terminator, pause site).

### The regulation summary diagram

**Figure 4:**
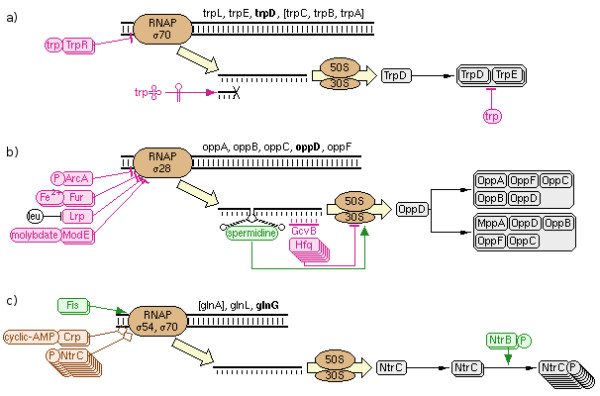
**Sample regulation summary diagrams for *E. coli *. a)** The trpD diagram shows inhibition by TrpR (a homodimer, as indicated by the appearance of multiple stacked copies) bound to tryptophan, attenuation by tryptophanyl-tRNA, and substrate-level regulation of the final complex by tryptophan. **b)** The oppD diagram shows inhibition by several transcription factors (the leucine ligand blocks regulation by Lrp), and regulation of translation by a spermidine riboswitch and RNA GcvB with accessory protein Hfq (a homosexamer). **c)** The glnG diagram shows activation and dual regulation by several transcription factors, and post-translational modification triggered by phosphorylated NtrB.

The Regulation Summary Diagram provides a compact and information-rich overview of all the direct regulatory influences on a single gene product, from transcription and translation to post-translational modifications and substrate-level modulation. Some examples of this diagram are shown in Figure 4. The diagram is organized according to biology’s central dogma, showing DNA being transcribed to RNA, and RNA being translated to protein. Regulation can occur at any stage along that path. At this level of detail, individual binding sites are not shown. Rather, activation or inhibition arrows are drawn pointing from transcription factors and their ligands to an icon representing RNA polymerase binding to the DNA strand. Attenuation regulators point to either a full-length or truncated mRNA strand. Translational regulators are shown binding to the mRNA strand and activating or inhibiting an icon representing the ribosome and translation. If the resulting polypeptide forms one or more complexes or participates in a reaction (such as a post- translational modification or sequestration interaction), the complexes or modified forms are also shown, along with any enzymes, activators or inhibitors that control the reaction. Finally, if some form of the protein has enzymatic activity, then its substrate-level activators or inhibitors are shown with arrows pointing to the enzyme. Mousing over any element of the diagram pops up a tooltip containing additional details or explanation.

### Pathway diagrams

The metabolic pathway diagrams generated by Pathway Tools can incorporate regulatory information. Pathway enzymes that are subject to regulation (whether at the substrate level or at the expression level) have a small plus or minus sign inside a circle next to their names. If the user passes the mouse over the icon, a tooltip appears indicating the regulator and the type of regulation. If an enzyme is regulated by some substrate in the same pathway, such as in the case of feedback inhibition, an arrow is drawn from the substrate to the enzyme it regulates. An example pathway that includes this kind of regulatory information can be found at http://biocyc.org/ECOLI/new-image?object=ARG%2bPOLYAMINE-SYN.

Signaling pathways consist of sets of reactions that form a regulatory cascade. Pathway Tools has specialized editing and display tools for signaling pathways. Signaling pathway diagrams are constructed manually by a curator. A number of two-component response regulator systems in E. coli and B. subtilis are represented as signaling pathways; an example is available at http://biocyc.org/ECOLI/new-image?object=PWY0-1493.

### Genetic regulation schematic

**Figure 5:**
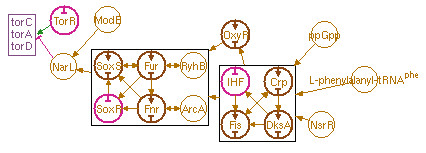
**The Genetic Regulation Schematic for the *E. coli *torCAD operon.**

The Genetic Regulation Schematic, depicted in Figure 5, shows both direct and indirect regulators that affect the expression (transcription and translation) of a gene or set of related genes. The diagram appears on both gene and pathway pages. To the left are one or more boxes representing the set of genes in an operon or pathway. Transcription factors or other entities that regulate a gene are drawn as circular icons, with arrows pointing to the regulated entities (arrows that represent direct regulation of the targeted genes are drawn in green or magenta, depending on whether they activate or inhibit expression, respectively; arrows that represent dual or indirect regulation are always drawn in brown). The expression of these transcription factors (or other protein or RNA regulators) may in turn be regulated by other factors (or they may regulate their own expression), and these interactions are also shown. Thus a network is built up that indicates the complete cascade of expression-level regulatory effects.

### Regulatory overview

The Regulatory Overview diagram presents a global picture of transcriptional regulation across the entire organism. Genes are clustered into groups on the basis of the set of transcription factors and sigma factors that regulate them, and arrows denoting regulatory interactions can be selectively drawn between genes of interest and the genes that regulate or are regulated by them (directly or indirectly, depending on the user’s preference).

**Figure 6:**
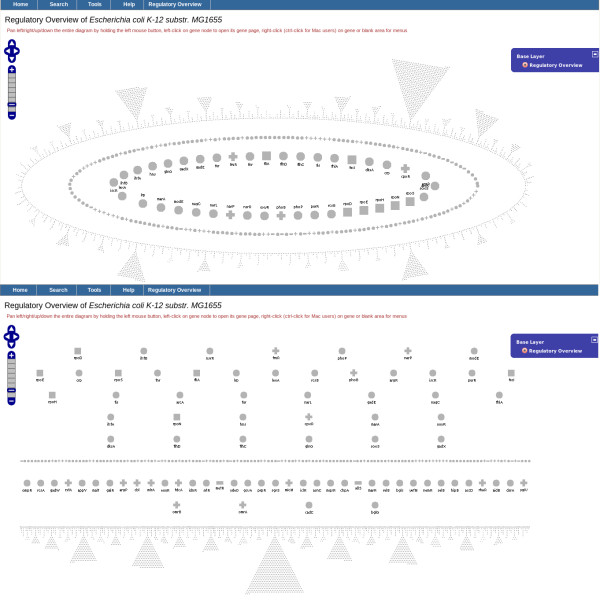
**Regulatory Overviews of *E. coli; * elliptical (upper) and layered (below) layouts are shown.** The user has not yet requested display of arrows that show regulatory relationships.

Two alternative layouts, shown in Figure 6, are supported. The upper elliptical layout shows all transcription factors and sigma factors in two inner ellipses, with all regulated genes that are not regulators in an outer ellipse. The lower, layered layout shows all transcription factors and sigma factors in the top layers, with all regulated genes that are not regulators in the lowest layer. The elliptical layout is designed to show the global regulatory network; the layered layout is designed to show smaller regulatory subnetworks.

**Figure 7:**
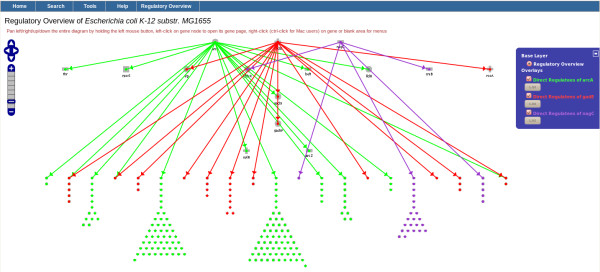
**A subnetwork using the layered layout.** This subnetwork was obtained by selecting three regulators (i.e., genes arcA, gadE, and nagC) and their direct regulatees.

A user can select a group of genes of interest for color-highlighting on the diagram. For example, the user could select the set of all genes in a given Gene Ontology category (e.g., all genes involved in cell division) A user could also select genes that are directly or indirectly regulated by a named gene, in which case arrows are drawn to indicate regulatory relationships among the selected genes. The set of highlighted genes can be redisplayed in its own page, using a layered or ellipse layout. This redisplay focuses on a regulatory subnetwork of selected genes to facilitate inspection of regulatory relationships among a smaller set of genes of interest. Figure 7 shows an example of a subnetwork created after the selection of three groups of genes based on regulatory relationships from three specific genes.

Users can color the Regulatory Overview diagram to show the results of a gene expression experiment in a regulatory context. This *Omics Viewer* mode can be used for all genes or for a subnetwork of genes to allow visual interrogation of the correlations among gene expression measurements and known regulatory interactions.

### Cellular overview

**Figure 8:**
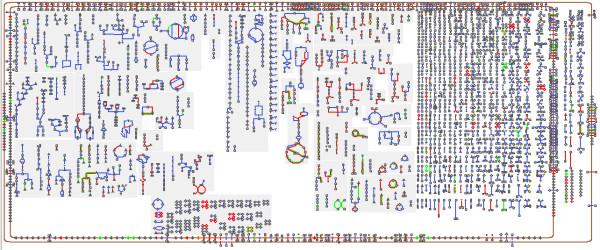
**The *E. coli *Cellular Overview diagram.** The CRP regulon is highlighted in red, and the IHF regulon is highlighted in green.

The Cellular Overview diagram depicts all pathways and metabolic and transport reactions that occur in an organism, and allows the user to visually explore the metabolic machinery controlled by different regulators. It is shown as a graph in which individual metabolites are the nodes with reactions forming the edges between them, and is organized into sections representing pathway classes. Transport reactions and membrane proteins are depicted around the diagram border, representing the cellular membrane. Various highlighting operations allow for visually querying this diagram — reaction arrows can be colored according to the enzymes (and their corresponding genes) that catalyze them. Several highlighting operations allow visualization of regulatory information (these operations are not yet available on the BioCyc Website, and require local installation of the Pathway Tools software). For example, one operation highlights all reactions whose enzymes are regulated at the substrate level by a specified compound. Another, shown in Figure 8, highlights all reactions whose genes are part of the regulon for (i.e., are regulated by) a specified transcription factor.

## Computational analyses using regulation data

This section describes components of Pathway Tools that analyze a collection of regulatory data to generate new biological insights. In addition, the Pathway Tools Application Programmer Interface (API) provides a rich set of operations to enable users to develop their own computational analyses, interacting with PGDB data via Lisp, Perl, Java, or our Web services interface [7].

Currently, analysis capabilities are limited because our ontology does not include quantitative data, nor does it describe how the effects of multiple regulatory elements combine. For example, if a protein has both an activator and an inhibitor, what is the effect if both are present? Does one override the other? Our model does not provide answers to these questions. Nonetheless, a variety of interesting qualitative deductions can be made with data encoded using our regulatory-interaction ontology.

### Object group operations and regulation-enrichment analysis

Suppose a scientist has identified a set of genes of interest — perhaps the genes behaved similarly in a gene-expression or other high-throughput experiment — and wants to find out more about how those genes are related. One reason a group of genes might behave similarly is if they are subject to similar regulatory influences. Thus, it is natural to ask what are the set of regulatory influences on a group of genes? Which genes not in the original set are also subject to the same regulatory influences, and what are the differences among them? Performing this kind of analysis is straightforward using the Pathway Tools Groups facility. A user can create a group of genes, for example, by uploading an omics dataset and selecting all genes whose expression level exceeds a threshold. Once the group has been specified, the user can ask for the complete set of regulators (transcriptional or translational) that regulate any gene in the group. The user can choose to retrieve only direct regulators of the gene group, or both direct and indirect regulators; the user can also extend the original group to include all genes in the same operon as the original genes. The user can proceed in the opposite direction, too — given the resulting set of regulators (or some subset of them), retrieve the complete set of regulated genes (again, either directly only, or both directly and indirectly). Or if only a handful of regulators seem to be relevant, the user can create groups of genes regulated by each, and then combine them by taking either the union or intersection. The user can then subtract the original group from the full set of regulated genes to obtain a comparison group. (Note that although all the base queries and transformations are available both through the BioCyc Website and on the locally installed software, the abilities to combine and subtract groups are currently available only by installing the software locally).

Another way to identify the most relevant set of regulators of a group of genes is to run a novel type of enrichment analysis that we have developed called regulation enrichment analysis. This analysis determines if a gene group is statistically enriched for containing genes regulated by certain regulators, relative to the complete set of genes and their regulators in the organism. As described above, the user can specify whether to consider direct regulation only, or both direct and indirect regulation. The regulation enrichment analysis can be used in isolation, or in combination with enrichment analysis for metabolic pathways and Gene Ontology terms. The results of the enrichment analysis form a new group of regulator genes that can be further manipulated in the manner described in the previous paragraph.

Additional group operations make it possible to determine the set of post-translational regulatory influences on a group of genes. A group of genes can be transformed to the group of proteins the genes code for. For that group of proteins, we can then ask what are their substrate-level activators, inhibitors, cofactors, and ligands. And for a group of metabolites, we can ask what are the set of proteins that bind the metabolites, or what are the set of enzymes the metabolites activate or inhibit.

These group operations could not be performed without the regulation ontology.

### Export to cytoscape

Given a set of regulatory interactions, we can create a directed graph representing the global regulatory network for an organism. In this graph, an edge from gene A to gene B means that gene A regulates gene B in some way. We have written software to generate such a graph containing all transcriptional and translational influences of one gene on another. Edges are labeled as either activating, inhibiting, dual (both activating and inhibiting, depending on context), or unknown regulatory effect. The graph can be used for analyses such as the one described in section Ranking genes according to regulatory influence below, or can be exported to XGMML [8] format, which can then be imported into Cytoscape [9]. Cytoscape is a generic network visualization package popular for displaying, visually querying, and analyzing (via plug-ins) biological networks. By exporting the regulatory network to Cytoscape, users gain access to a number of third party Cytoscape plug-ins (e.g. [10,11]) that analyze regulatory networks in the context of experimental data such as expression data.

### Ranking genes according to regulatory influence

How can we assess the degree of regulatory influence of each gene within the regulatory network? Although lacking quantitative information, we posit that the influence of a gene is proportional to the number of genes it directly regulates and, to a lesser extent that falls off with number of intervening steps, to the number of genes that it indirectly regulates. In addition, if a gene is the sole regulator of some other gene, its influence is likely to be greater than if it is just one of many regulators for that gene. We can compute an influence score for every gene in the regulatory network using a simplified version of the approach used in Google’s PageRank algorithm [12].

Starting with the regulatory graph described in Section Export to cytoscape, we construct an adjacency matrix such that element (i,j) of the matrix is 1 if gene i regulates gene j and 0 otherwise. The influence score for each gene can then be obtained by computing the dominant eigenvector of the matrix using the Power Method. Table 3 lists the 20 highest scoring genes in EcoCyc [3] and BsubCyc computed using this method for a graph that contains transcription, translation and sigma-factor regulation, but not any regulation due to post-translational modifications. Scores are normalized to give the highest-scoring gene a score of 100. The Direct Targets column lists the number of genes directly regulated by the regulator. A similar table can be generated for any database that contains regulation data.

**Table 3:** The 20 genes in EcoCyc and BsubCyc with the highest influence scores

***E. coli *K-12**				***B. subtilis ***			
**Gene**	**Product Role**	**Score**	**Direct Targets**	**Gene**	**Product Role**	**Score**	**Direct Targets**
rpoD	sigma-70 factor	100	2282	sigA	sigma-43 factor	100	863
rpoE	sigma-24 factor	44	550	sigB	sigma-37 factor	75	101
crp	cAMP receptor protein	36	465	spo0A	response regulator	55	88
lexA	SOS response transcriptional repressor	32	56	sigE	sporulation-specific sigma-29 factor	51	128
ihfA	integration host factor, *α*-subunit	26	225	spoIVCB	sporulation-specific sigma-K factor precursor (N-terminal half)	46	101
ihfB	integration host factor, *β*-subunit	26	225	spoIIIC	sporulation-specific sigma-K factor precursor (C-terminal half)	46	101
rpoS	sigma-S factor	26	316	spoIIID	sporulation-specific transcriptional regulator	46	37
rpoH	sigma-32 factor	25	450	gerE	transcriptional regulator	34	104
cpxR	stress response regulator	23	58	abrB	regulator of transition state genes	25	100
fis	factor for inversion stimulation	19	223	sigH	sigma-30 factor	22	21
gadX	acid resistance system regulator	14	28	rok	repressor of comK	3	27
fur	ferric uptake regulator	13	131	comK	competence transcription factor	3	55
arcA	anaerobic response regulator	11	160	degU	two-component response regulator	1.6	19
hns	histone-like nucleoid structuring protein	11	174	codY	GTP and BCAA-dependent transcriptional regulator	1.6	63
fnr	aerobic to anaerobic transition regulator	10	294	sinR	post-exponential phase response regulator	1.6	21
dnaA	chromosomal replication initiator protein	8.2	12	med	positive regulator of comK	1.6	1
cytR	cytidine repressor	8.2	12	scoC	extracellular protease production and sporulation regulator	0.8	16
nsrR	nitrite-sensitive repressor	6.8	83	senS	transcriptional activator	0.8	2
rpoN	sigma-54 factor	6.4	179	salA	Mrp family regulator	0.4	1
gadE	acid resistance system regulator	5.4	36	sigG	sporulation-specific sigma-G factor	0.4	84

### Inferring regulatory influences on a gene

If we want to determine the overall effect of a regulator on a gene, we need to construct a different graph, one that considers the parity of the regulation (activating or inhibiting) and includes all possible ways by which one entity can regulate another. We have written such a program^b^ that constructs the graph of all factors that unambiguously positively or negatively affect a given gene product, either directly or indirectly. Effects are considered to be multiplicative in terms of their parity – if *A* inhibits *B*, and *C* inhibits *A*, then we consider *C* an indirect activator of *B*. If *A* activates *B* by one path and inhibits *B* by another, then in the absence of any quantitative information, we cannot determine the overall effect (if any) of *A* on *B*; accordingly, we declare its regulatory effect as unknown and further regulatory effects on *A* are ignored (alternative hypotheses are that the overall effect of *A* on *B* is determined by the shortest path from *A* to *B* when paths are of different lengths, or by a majority rules test when more activating than inhibiting paths exist or vice versa; we have not implemented any of these alternatives).

This algorithm takes a very broad view of regulation; in addition to transcription factors, translational regulators and substrate-level modulators, we also consider sigma factors and producing/consuming reactions to be regulating influences. If an entity is product of a reaction, then both the reactants and the enzyme of the reaction are considered activators of the entity. If an entity is a reactant in a reaction, then the enzyme and any other reactants are considered inhibitors of the entity (because they promote its consumption). Reactions that can proceed in either direction are ignored for the purpose of this analysis. Reactions of substrates that participate in large numbers of reactions are also ignored because we consider them unlikely to be used for regulatory purposes, and omitting them simplifies the graph.

**Figure 9:**
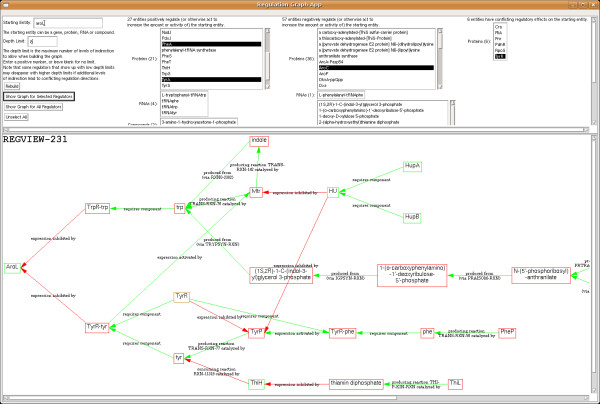
**A graph showing how the *E. coli *AroL protein is regulated by selected regulators.**

Once the complete graph for a given protein has been constructed, it can be queried to generate a list of entities that either positively or negatively regulate the protein (the list may be incomplete because of regulators with conflicting influences). Given a particular regulator, the graph can be queried to determine the path(s) through which it influences the original entity. For example, Figure 9 shows a partial graph for the *E. coli* AroL protein. The top pane contains the full list of regulators whose effect is known for the specified depth limit. The bottom pane shows how the target is regulated by the selected regulators, with entities whose overall effect is positive shown in green, those whose overall effect is negative shown in red, and those whose effect is unknown shown in brown. The graph can be read from left to right. For example, expression of AroL is inhibited by both the the TrpR transcription factor bound to tryptophan and the TyrR transcription factor bound to tyrosine. Both of these transcription factors require their respective ligands for these activities, although the effect of TyrR without its ligand is ambiguous. Reactions that produce or consume tryptophan and tyrosine are then subject to further regulation.

## Related work

Most other databases containing information on biological regulation specialize in one or a few specific types of regulation — none attempt to cover the full range of regulatory interactions as Pathway Tools does. Other databases that contain regulatory network information include DBTBS [6], RegTransBase [13], TRANSFAC [14], CoryneRegNet [15], ProdoNet [16], TransmiR [17], and YEASTRACT [18]. Most of these databases contain information on transcription-factor-based regulation only. RegulonDB [5] contains transcription-factor-based data as well as RNA-based regulation such as information on riboswitches, attenuation and small RNA regulators. BRENDA [19] contains extensive data on enzyme activators and inhibitors.

Most of the databases listed above offer visualizations similar to our transcription unit diagram. CoryneRegNet, ProdoNet and RegulonDB also include a network-based diagram, similar to a subset of our Regulatory Overview Diagram. CoryneRegNet provides the ability to display omics data on its regulatory network diagrams, as well as a plug-in, CoryneRegNetLoader [10], capable of importing its data into Cytoscape where the entire network can be visualized and analyzed. We know of no other software tool or database that is capable of generating anything similar to our Regulation Summary Diagram.

There exist a variety of analytical tools for regulatory networks. BioQuali [11] and COMA [10] attempt to validate regulatory networks against gene expression datasets and point out inconsistencies or suggested changes. While Pathway Tools has no similar capability, BioQuali and COMA are both implemented as Cytoscape plug-ins, and therefore can accept a Pathway Tools-generated regulatory network as input. Other tools, such as DEGAS [20] and KeyPathwayMiner [21], use omics data to mine protein interaction networks and attempt to infer regulatory sub-networks. While these tools can be considered somewhat analogous to our enrichment analysis, their approach is very different.

## Conclusions

Pathway Tools provides the ability to represent and capture a wide range of regulatory data. It differs from the other software and database environments in the wider range of regulatory interactions that it supports, in the greater number of tools that it affords to manipulate those interactions, and in the value it adds by integrating different types of regulatory data with one another. Regulatory data can also be integrated with the reaction, pathway and genomic data that Pathway Tools provides. It is this integration that allows Pathway Tools to show regulatory information on pathway diagrams, and to build visualizations such as the Regulation Summary Diagram, which combines many disparate types of information.

## Availability and requirements

**Project name:** Pathway Tools

**Project home page:**http://bioinformatics.ai.sri.com/ptools/

**Operating system(s):** MacOS, Windows, Linux

**Programming language:** Common Lisp

**License:** Free to academics; includes source code with limited rights to redistribute

**Any restrictions to use by non-academics:** Fee required

## Endnotes

^a^The term transcription unit refers to a set of one or more genes transcribed together from one promoter — the term operon implies more than one gene.^b^The tool described in this section is a prototype that is not currently a part of Pathway Tools.

## Competing interests

The authors benefit financially from commercial licensing of the Pathway Tools software.

## Authors’ contributions

SP wrote the majority of the manuscript; ML and PK also wrote parts of the manuscript. SP implemented the transcription unit diagram, regulation summary diagram, pathway diagrams, genetic-regulation schematic, Cellular Overview, and computational analyses of regulatory data. ML implemented the Regulatory Overview. PK supervised the development and implementation of the software. All authors read and approved the final manuscript.
